# Changes in oxygen delivery during experimental models of cerebral malaria

**DOI:** 10.1016/j.exppara.2023.108608

**Published:** 2023-09-05

**Authors:** Vinay P. Jani, Alexander T. Williams, Leonardo Carvalho, Pedro Cabrales

**Affiliations:** a Department of Bioengineering, University of California, San Diego, La Jolla, CA, 92093-0412, USA; b Instituto Oswaldo Cruz, Fio Cruz Rio de Janeiro, Brazil; c La Jolla Bioengineering Institute, 505 Coast Boulevard South, Suite 406, La Jolla, CA, 92037, USA

**Keywords:** Cerebral malaria, Phosphorescence quenching microscopy, Microcirculation, Hemodynamics, Oxygen affinity

## Abstract

Cerebral malaria (CM) is a severe manifestation of malaria that commonly occurs in children and is hallmarked by neurologic symptoms and significant *Plasmodium falciparum* parasitemia. It is currently hypothesized that cerebral hypoperfusion from impaired microvascular oxygen transport secondary to parasitic occlusion of the microvasculature is responsible for cerebral ischemia and thus disease severity. Animal models to study CM, are known as experimental cerebral malaria (ECM), and include the C57BL/6J infected with Plasmodium berghei ANKA (PbA), which is ECM-susceptible, and BALB/c infected with PbA, which is ECM-resistant. Here we sought to investigate whether changes in oxygen (O_2_) delivery, O_2_ flux, and O_2_ utilization are altered in both these models of ECM using phosphorescence quenching microscopy (PQM) and direct measurement of microvascular hemodynamics using the cranial window preparation. Animal groups used for investigation consisted of ECM-susceptible C57BL/6 (Infected, n = 14) and ECM-resistant BALB/c (Infected, n = 9) mice. Uninfected C57BL/6 (n = 6) and BALB/c (n = 6) mice were included as uninfected controls. Control animals were manipulated in the exact same way as the infected mice (except for the infection itself). C57BL/6 ECM animals at day 6 of infection were divided into two cohorts: Early-stage ECM, presenting mild to moderate drops in body temperature (>34 < 36 °C) and Late-stage ECM, showing marked drops in body temperature (<33 °C). Data taken from new experiments conducted with these animal models were analyzed using a general linear mixed model. We constructed three general linear mixed models, one for total O_2_ content, another for total O_2_ delivery, and the third for total O_2_ content as a function of convective flow. We found that in both the ECM-susceptible C57BL/6J model and ECM-resistant BALB/c model of CM, convective and diffusive O_2_ flux along with pial hemodynamics are impaired. We further show that concomitant changes in p50 (oxygen partial pressure for 50% hemoglobin saturation), only 5 mmHg in the case of late-stage CM C57BL/6J mice, and O_2_ diffusion result in insufficient O_2_ transport by the pial microcirculation, and that both these changes are required for late-stage disease. In summary, we found impaired O_2_ transport and O_2_ affinity in late-stage ECM, but only the former in either early-stage ECM and ECM-resistant strains.

## 1. Introduction

Cerebral malaria (CM) is one manifestation of malaria in the pediatric populace in areas where the parasite is endemic (Idro et al., 2005). Often the diagnosis is clinical and involves deep coma and significant *P. falciparum* parasitemia. Symptoms accompanying CM include headache, stiff neck, drowsiness, disorientation, and seizures. CM also often presents with several metabolic acidosis, accompanied by abnormal respiratory patterns. Morbidity and mortality are high with 11% experiencing neurologic sequelae and between 13 and 25% of cases leading to death (Idro et al., 2005; Idro, 2003; Maitland and Marsh, 2004). The exact pathology of CM is unknown, though postmortem CM studies have revealed that parasitized red blood cells (pRBCs), particularly sequestrations of trophozoites and schizonts, adhere to the capillary endothelium thereby resulting in obstruction of the pial microvasculature (Beare et al., 2009; Pongponratn et al., 1991). Many pathophysiological aspects of CM are still not completely understood. Possible contributing mechanisms involve microcirculatory dysfunction, vasoconstriction, hemolysis, and reduced deformability of red blood cell (RBC). These mechanisms are not mutually exclusive, but they all result in impaired blood flow. It is hypothesized that these microvascular obstructions in conjunction with anemia result in depressed cerebral oxygenation and subsequently cerebral ischemia, coma, and death, if left untreated (Beare et al., 2009; Pongponratn et al., 1991). Clinically cerebral hypoperfusion and ischemia are assessed indirectly by elevated cerebrospinal fluid lactate and retinal hypoperfusion (White et al., 1985; MacPherson et al., 1985; Beare et al., 2006). Susceptible murine models of experimental cerebral malaria (ECM) by *Plasmodium berghei* ANKA (PbA) have further supported the hypothesis of cerebral hypoperfusion and ischemia by demonstrated increased expression of hypoxia-inducible factor-1α (HIF-1α), and positive staining by the hypoxia probe (pimonidazole) (Cabrales et al., 2013).

Beyond microvascular occlusion, O_2_ transport is mediated by blood pH (Bohr effect), hemoglobin (Hb) concentration (anemia), and core body temperature, all of which are altered systemically during malarial infection. Particularly in CM, intra-erythrocytic parasitic digestion of Hb into hemozoin further contributes to anemia. We have previously reported oxygen tension in the cerebral pial circulation of two strains of mice, namely the C57BL/6J, which is ECM-susceptible, and BALB/c, which is ECM-resistant (Cabrales et al., 2013). Specifically, we demonstrated impaired pial microvascular hemodynamics and depressed oxygen tension from phosphorescence quenching microscopy (PQM) (Cabrales et al., 2013; Tsai et al., 1998, 2003; Sharan et al., 2008; Vovenko, 1999, 2009) in the ECM-susceptible C57BL/6J strain compared to the BALB/c ECM-resistance strain (van Santbrink et al., 1996). However, these previous studies did not simultaneously acquire perivascular and intravascular O_2_ tensions, limiting quantification of convective and radial/diffusive O_2_ flux, O_2_ consumption, and more sophisticated measures of pial microvascular hemodynamics. Here, we sought to expand upon our previous findings by investigating whether O_2_ delivery, O_2_ flux, or O_2_ utilization is altered and to identify whether increased pial oxygen consumption or impaired delivery is the mechanism for depressed pial tissue oxygenation. Using PQM and ECM resistant and susceptible animal models, we demonstrate impaired microvascular O_2_ delivery and utilization secondary to CM infection independent of parasitic load. Furthermore, by examining microvascular O_2_ content and delivery as a function of both vessel diameter and convective flow, we demonstrate impaired axial and radial flux, further supportive of impaired O_2_ diffusion and altered O_2_ metabolism in both models. Unique to this study is paired acquisition of perivascular and intravascular O_2_ tension in two strains of mice (namely C57BL/6J and BALB/c) with sophisticated statistical modeling for calculation of O_2_ delivery, flux, and utilization.

## 2. Methods

### Closed cranial window animal preparation.

Animal handling and care followed the NIH Guide for Care and Use of Laboratory Animals. All protocols were approved by the La Jolla Bioengineering Institutional Animal Care and Use Committee. 8 to 10-week old C57Bl/6J and Balb/cJ mice (Jackson Laboratories, ME) were implanted with a closed cranial window model as described elsewhere (Mostany and Portera-Cailliau, 2008). Briefly, mice were anesthetized with ketamine-xylazine and were administered dexamethasone (0.2 mg/kg), carprofen (5 mg/kg) and ampicillin (6 mg/kg) subcutaneously, to prevent post-surgical swelling of the brain, inflammatory response, and infection. After shaving the head and cleansing with ethanol 70% and betadine, the mouse was placed on a stereotaxic frame and the head immobilized using ear bars. The scalp was removed with sterilized surgical instruments and lidocaine-epinephrine was applied on the periosteum, which was then retracted to expose the skull. A 3–4 mm diameter skull opening was made in the left parietal bone using a surgical drill. Under a drop of saline, the craniotomy was lifted away from the skull with very thin-tip forceps and gelfoam previously soaked in saline was applied to the dura mater to stop any eventual small bleeding. The exposed area was covered with a 5 mm glass cover slip secured with cyanocrylate-based glue and dental acrylic. Carprofen and ampicillin were given daily for 3–5 days after recovery from surgery. Mice presenting signs of pain or discomfort were euthanized with 100 mg/kg of euthasol IP. Two to three weeks after surgery, mice fulfilling the inclusion criteria (see below) were inoculated with P. berghei ANKA and, on day 6 of infection, they were lightly anesthetized with isoflurane (4% for induction, 1–2% for maintenance) and held on a stereotaxic frame for measurements of pH and pO_2_.

### Inclusion criteria.

Animals were suitable for the experiments if: 1) animal behavior was normal and 2) microscopic (x350 magnification) examination of the cranial window did not reveal signs of edema or bleeding.

### Parasite infection.

Animals were inoculated with an IP injection of 1 × 106 Plasmodium berghei ANKA parasites expressing the green fluorescent protein (PbA-GFP, a donation from the Malaria Research and Reference Reagent Resource Center – MR4, Manassas, VA; deposited by CJ Janse and AP Waters; MR4 number: MRA-865). Parasitemia, body weight, rectal temperature and clinical status (using six simple tests adapted from the SHIRPA protocol, as previously described) were monitored daily from day 4 of the infection (Lackner et al., 2006). Parasitemia was checked using flow cytometry by detecting the number of fluorescent GFP-expressing pRBCs in relation to 10,000 RBCs. ECM was diagnosed when one or more of the following clinical signs of neurological involvement were observed: ataxia, limb paralysis, poor righting reflex, seizures, roll-over, or coma.

### Physiological ranges of the variables measured for the animal species used.

Two groups of animals, C57BL/6 (n = 6) and BALB/c (n = 6), instrumented with the closed cranial window were used to characterize normal microhemodynamic (vessel diameter and blood flow), intravascular and perivascular PO2s and pH in the pial microenvironment.

### Experimental Groups.

Group 1 aimed to establish the effects of PbA infection in microhemodynamics, intravascular and perivascular PO2s and pH in the pial microenvironment. The group consisted of ECM-susceptible C57BL/6 (Infected, n = 14) and ECM-resistant BALB/c (Infected, n = 9) mice. Uninfected C57BL/6 (n = 6) and BALB/c (n = 6) mice were included as controls. Control animals were manipulated in the exact same way as the infected mice (except for the infection itself). C57BL/6 ECM animals at day 6 of infection were divided in two cohorts: Early-stage ECM, presenting mild to moderate drops in body temperature (>34 < 36 °C) and Late-stage ECM, showing marked drops in body temperature (<33 °C). Another group, Group 2, was included to establish the relation between vascular inflammation resulting from PbA infection and microhemodynamics and oxygenation in relation to ECM pathophysiological changes. The group consisted of C57BL/6 (Infected, n = 9) mice to which leukocyte adhesion, blood flow and PO2 levels were measured. Similarly, as in Group 1, the ECM animals at day 6 of infection were divided in two cohorts: Early-stage ECM and Late-stage ECM. All experiments were repeated at least once.

### Experimental Setup.

Animals were lightly anesthetized with isoflurane (4% for induction, 1–2% for maintenance). They were secured to the microscopic stage of an intravital microscope (BX51WI, Olympus, New Hyde Park, NY) on a stereotaxic frame with the head gently held with ear bars for epi-illumination imaging. Body temperature, measured pre-anesthesia, was maintained with a heating pad. The tissue image was projected onto a charge-coupled device camera (COHU 4815) connected to a videocassette recorder and viewed on a monitor. Measurements were carried out using a 40X (LUMPFL-WIR, numerical aperture 0.8, Olympus) water immersion objective. The animals did not recover from anesthesia, as they were euthanized (Euthasol 100 mg/kg, IP) right after the intravital microscopy measurements.

### Microhemodynamics.

A video image-shearing method was used to measure vessel diameter (D) (Intaglietta and Tompkins, 1973). Changes in arteriolar and venular diameter from baseline were used as indicators of a change in vascular tone. Arteriolar and venular centerline velocities were measured on-line using the photodiode cross-correlation method (Photo Diode/Velocity Tracker Model 102B, Vista Electronics, San Diego, CA). The measured centerline velocity (V) was corrected according to vessel size to obtain the mean RBC velocity (Lipowsky and Zweifach, 1978; Lipowsky et al., 1978). Blood flow (Q) was calculated from the measured values as Q = π × V (D/2)^2. This calculation assumes a parabolic velocity profile and has been found to be applicable to tubes of 15–80 μm internal diameters and for Hcts in the range of 6–60% (Lipowsky and Zweifach, 1978; Lipowsky et al., 1978).

### Microvascular PO2 distribution.

High resolution non-invasive microvascular pO_2_ measurements were made using phosphorescence quenching microscopy (PQM) (Golub and Pittman, 2008). PQM is based on the relationship between the decay rate of excited Palladium-mesotetra-(4-carboxyphenyl) porphyrin (Frontier Scientific Porphyrin Products, Logan, UT) bound to albumin and the O_2_ concentration according to the Stern-Volmer equation (Golub and Pittman, 2008; Kerger et al., 2003). The method was used previously in microcirculatory studies to determine pO_2_ levels in different tissues (Kerger et al., 2003). pO_2_. measurements by PQM were obtained following these steps for all groups: 1) the probe was injected (tail injection of 15 mg/kg at a concentration of 10 mg/ml of the phosphorescence complex 10 min before O_2_ measurements); 2) the tissue was illuminated (pulsed light at 420 nm wavelength) to excite the probe into its triplet state; 3) the emitted phosphorescence (680 nm wavelength) was collected and analyzed to yield the phosphorescence lifetime; and 4) the phosphorescence lifetime was converted into O_2_ concentration, pO_2_. The phosphorescence lifetimes are concentration independent, which permit extravascular fluid pO_2_ measurements, although the dye albumin complex that extravasates is very small. Extravascular fluid pO_2_ was measured in regions in between functional capillaries. PQM allows for precise localization of the pO_2_ measurements without subjecting the tissue to injury. These measurements provide a detailed understanding of microvascular O_2_ distribution and indicate whether O_2_ is delivered to the interstitial areas.

### Hematocrit and hemoglobin.

Blood was collected from the tail in heparinized glass capillaries. Hemoglobin was determined spectrophotometrically from a single drop of blood in a B-Hemoglobin analyzer (Hemocue, Stockholm, Sweden). Hematocrit was estimated by centrifugation.

### Oxygen delivery and extraction.

Oxygen delivery, DO_2_ was approximated as

(1) $${DO}_{2}=\left[\left({RBC}_{Hb}\times \gamma \times S_{A}\right)\right]\times Q_{A}$$

where RBC_Hb_ is the total Hb (g/dL), *γ* is the O_2_ carrying capacity of saturated Hb, approximated as 1.34 mL O_2_/g Hb, S_A_ is arteriolar blood oxygen saturation, and Q_A_ is arteriolar flow. The relationship between oxygen delivery and flow, experimentally determined here, reflects changes in arteriolar blood oxygen saturation and the oxygen carrying capacity of saturated Hb, both known to be altered by malaria.

Similarly, arterio-venous oxygen extraction (O_2_ A-V Extraction, or VO_2_) was approximated as

(2) $$DV=\left[\left({RBC}_{Hb}\times \gamma \times S_{A-V}\right)\right]\times Q_{A-V}$$

where S_A-V_ is the difference between arteriolar and venular oxygen saturation and Q_A-V_ is the average of arteriolar and venular flow rate. O2 saturations were approximated using the blood O_2_ equilibrium curve.

### Statistical Analysis.

Results are presented as mean ± standard deviation. Data between groups was analyzed using a non-parametric Kruskal-Wallis test. When appropriate, post hoc analyses were performed with the Dunns’ multiple comparison test. Microvascular O_2_ content and O_2_ delivery were compared using a general linear mixed model as a function of either diameter or flow. All these measurements are experimental. For analysis, data were linearized by log-log transformation to allow for analysis. Differences in radial and axial O_2_ fluxes were compared using an analysis of covariance (ANCOVA) from the aforementioned general linear mixed model. The only modeled data in the results presented are fluxes from linear modeling of experimental data. All statistics were calculated using GraphPad Prism 9.1.2. Changes were considered statistically significant if p < 0.05.

## 3. Results

### Systemic Blood Gas Parameters.

No significant differences were observed in hematocrit, arterial blood pH, blood oxygen affinity (p50), and body temperature between C57BL/6J and BALB/c mice at baseline, though were altered with infection at day 6. These results along with detailed statistical analysis are shown in Table 1.

### Diffusive Oxygen Transport and Delivery.

**Fig. 1.**
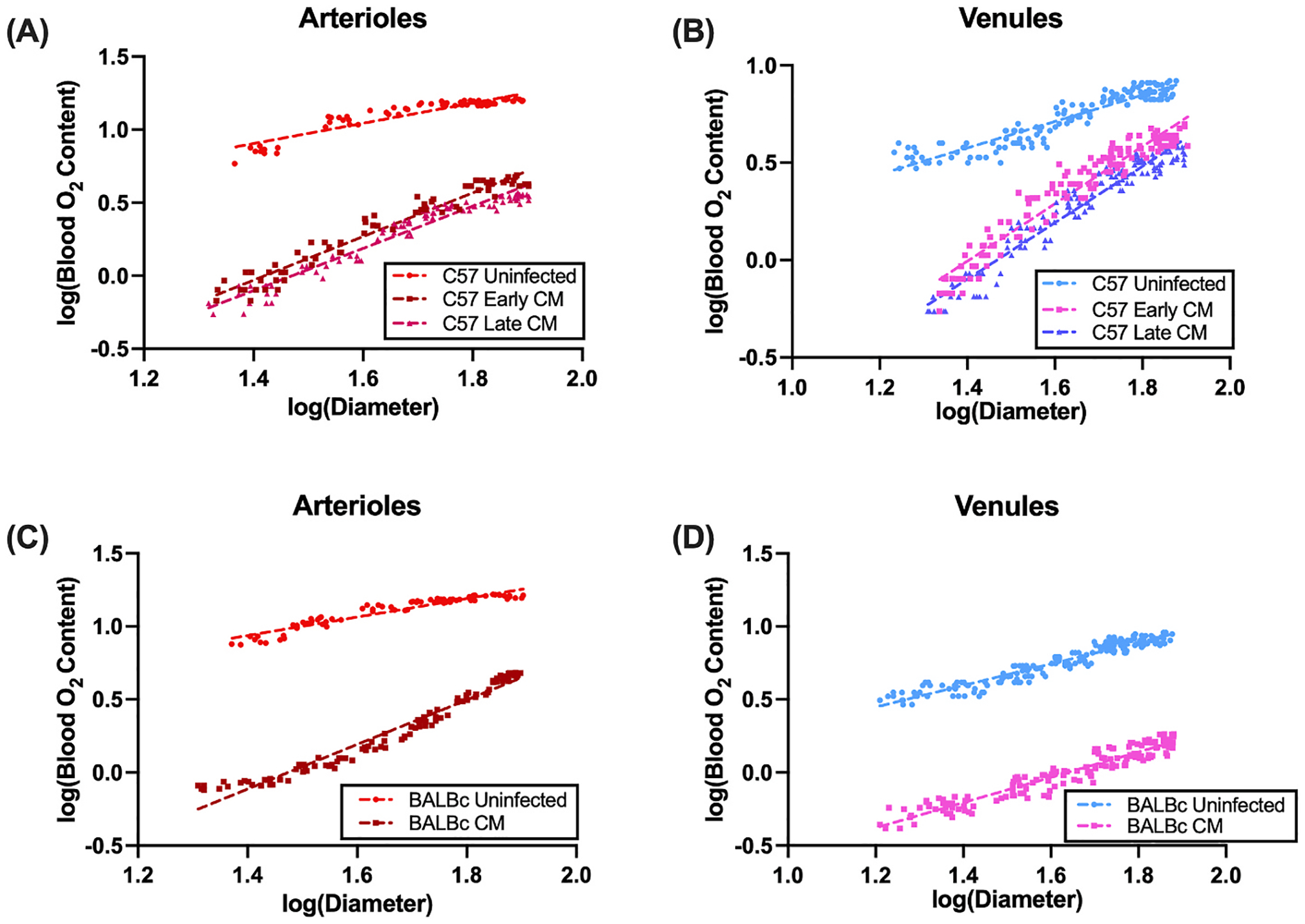
Log-log plots of Blood O_2_ Content vs Diameter from PQM show impaired diffusive flux. Shown here are log-log plots for (A) Arterioles and (B) Venules from C57 ECM-susceptible mice and (C) Arterioles and (D) Venules from BALB/c ESM-resistant mice. Linear regression lines are shown, with the inverse of the slope corresponding to diffusive flux. Data were analyzed using a general linear mixed model.

We first sought to quantify the effect of CM infection in both the ECM-susceptible C57BL/6J strain and the ECM-resistant BALBc strain. Total arteriolar and venular oxygen content were quantified using equation (1) from PQM data. Total O_2_ content was next analyzed as a function of diameter. Log-log plots with corresponding linear regression analysis are shown in Fig. 1. To quantify changes between uninfected and CM infection, we used a general linear model, log(total O_2_ content) ~ *β*_0_ + *β*_1_ log(D) + *β*_2_ [group] + *β*_3_ [log(D):group]. Here, log(D) corresponds to the log of diameter, [group] corresponds to the group (uninfected, early CM, and late CM in the case of the C57BL/6J and uninfected and CM in the case of BALBc), and [log(D):group] corresponds to the interaction term. We took 1/*β*_1_ to reflect radial diffusive flux, which is the inverse of the slope of the log-log plots shown in Fig. 1, *β*_2_ to reflect the absolute difference in total O_2_ content between groups, and *β*_3_ to represent whether infection alters radial diffusive flux. In arterioles from the C57BL/6J strain (Fig. 1A), we observed a significant depression in total oxygen content (p = 1.09 × 10^−8^) and radial diffusive flux (p = 1.0 × 10^−15^, interaction p = 0.00135) in both CM groups compared to uninfected controls. We observed a similar result in venules from this strain (*β*_1_ p = 1.0 × 10^−15^, *β*_2_ p = 1.0 × 10^−15^, interaction p = 1.0 × 10^−15^). For the ECM-resistant BALBc strain, in arterioles (Fig. 1C), we also observed a depressed total oxygen content (p = 1.0 × 10^−15^) and depressed radial diffusive flux (p = 1.0 × 10^−15^, interaction p = 1.0 × 10^−15^) between the CM group and uninfected controls. We observed similar results for venules (*β*_1_ p = 1.0 × 10^−15^, *β*_2_ p = 1.0 × 10^−15^, interaction p = 8.55 × 10^−6^). Log-log plots for this analysis are shown in Fig. 1D.

**Fig. 2.**
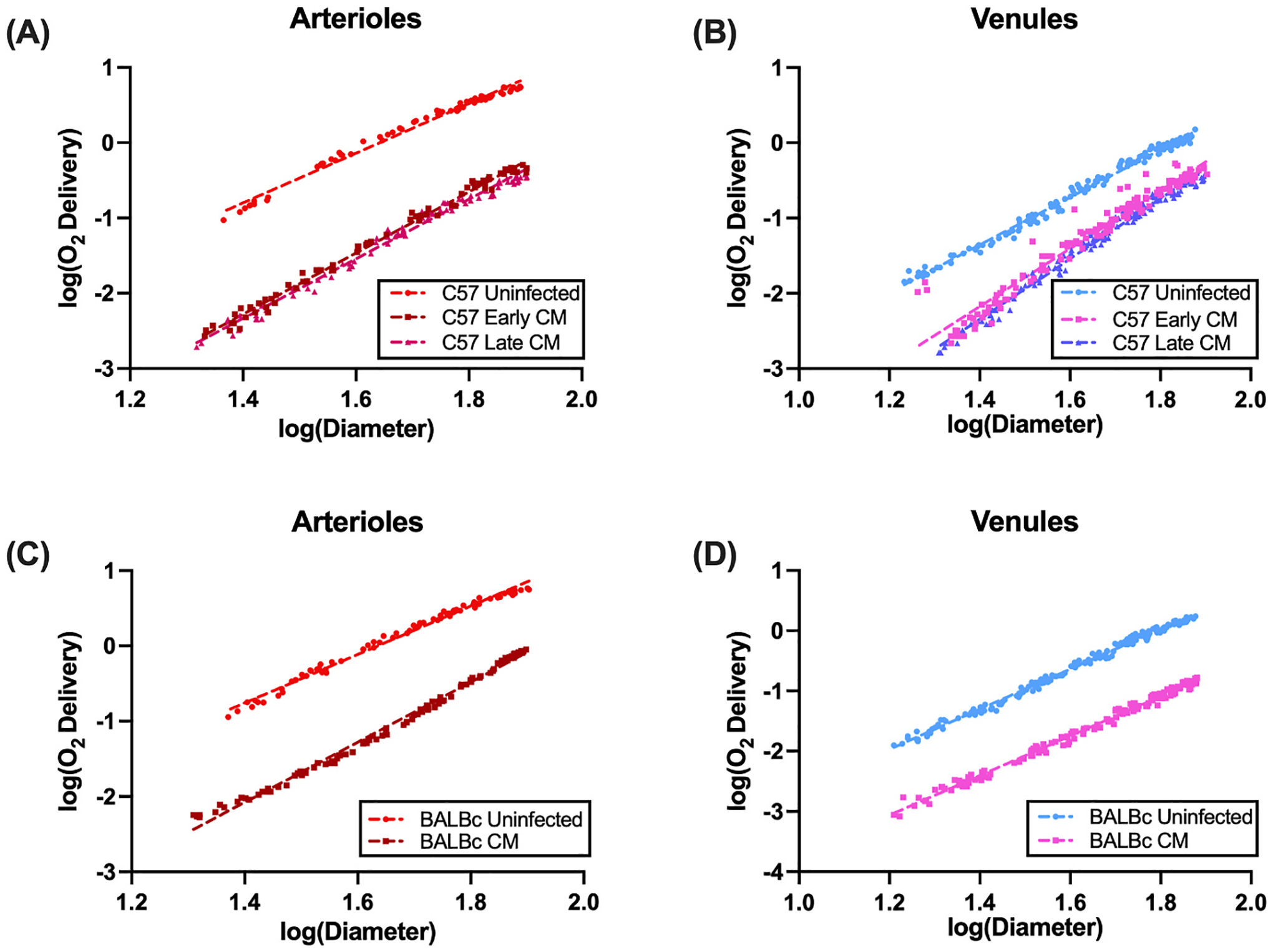
Log-log plots of Blood O_2_ Content vs Diameter from PQM show impaired consumption. Shown here are log-log plots for (A) Arterioles and (B) Venules from C57 ECM-susceptible mice and (C) Arterioles and (D) Venules from BALB/c ESM-resistant mice. Linear regression lines are shown, with the inverse of the slope corresponding to consumption. Data were analyzed using a general linear mixed model.

The O_2_ content only reflects the concentration gradient for diffusion but does not reflect changes in consumption or metabolism. To test whether CM infection in both the ECM-susceptible C57BL/6J strain and ECM-resistant BALBc strain altered oxygen consumption, we calculated O_2_ delivery (DO_2_) using equation (2). Log-log plots for arterioles and venules from both strains are summarized in Fig. 2. As before, to quantify changes post CM infection, we used a general linear model, namely log(DO_2_) ~ *β*_0_ + *β*_1_ log(D) + *β*_2_ [group] + *β*_3_ [log(D):group]. Here, we took 1/*β*_1_ to represent total O_2_ consumption, with *β*_2_ and *β*_3_ having the same interpretation as above. We found that in arterioles from the ECM-susceptible C57BL/6J strain that O_2_ delivery was depressed (p = 3.68 × 10^−8^) in both early and late CM compared to uninfected controls. Consumption, though depressed compared to uninfected controls in both CM groups (p = 1.0 × 10^−15^) was not altered by infection (interaction p = 0.3160). These results, along with corresponding results from venules are summarized in Fig. 2A and B. We next assessed consumption in the ECM-resistance BALBc strain. We found depressed DO_2_ and depressed consumption for arterioles (*β*_1_ p = 1.0 × 10^−15^, *β*_2_ p = 1.0 × 10^−15^, interaction p = 1.0 × 10^−15^) and venules (*β*_1_ p = 1.0 × 10^−15^, *β*_2_ p = 1.0 × 10^−15^, interaction p = 0.172). In the latter, however, the change in consumption was directly a result of the depression in DO_2_. These results are summarized in Fig. 2C and D.

### Convective Oxygen Transport.

**Fig. 3.**
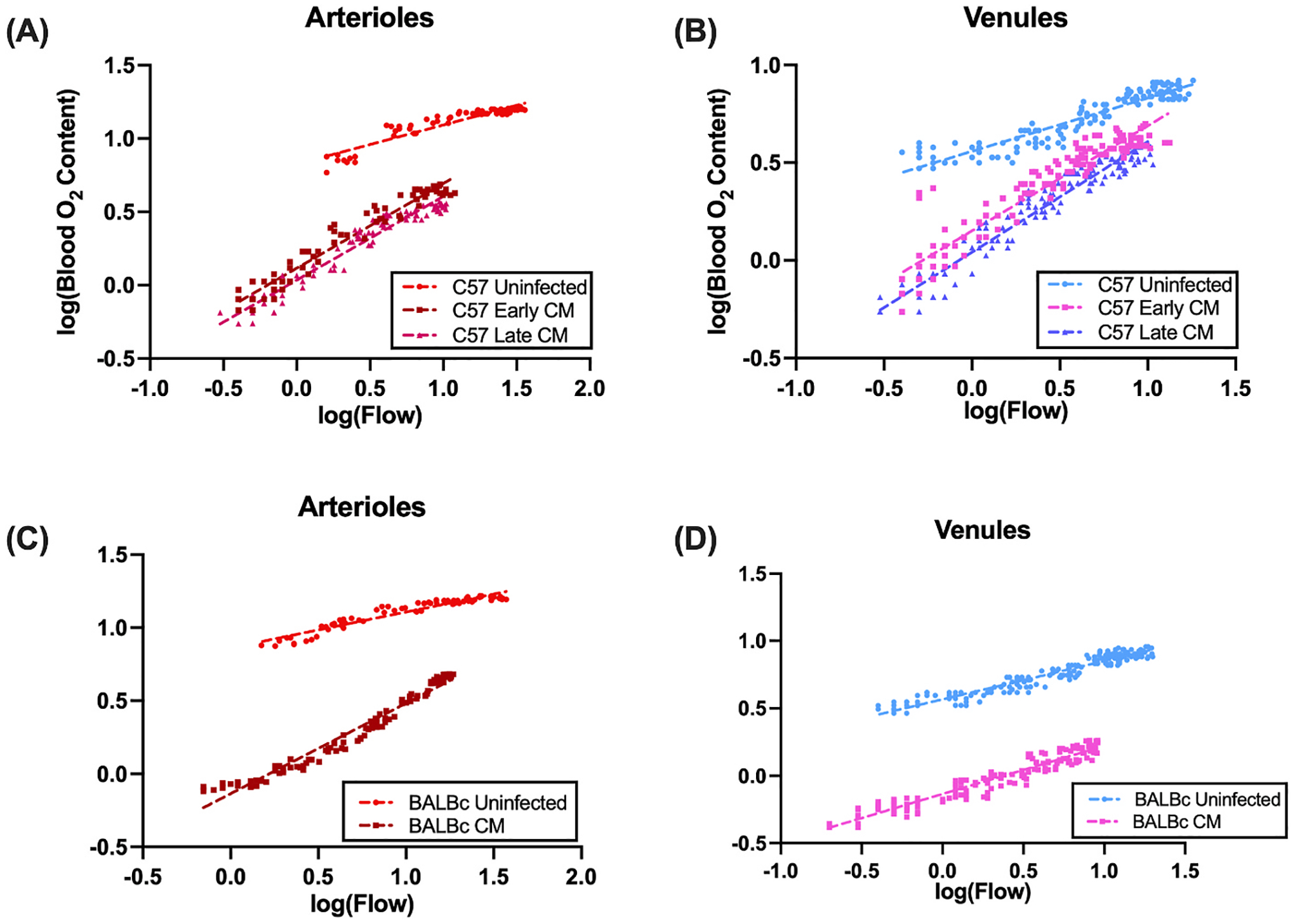
Log-log plots of Blood O_2_ Content vs Diameter from PQM show convective flux. Shown here are log-log plots for (A) Arterioles and (B) Venules from C57 ECM-susceptible mice and (C) Arterioles and (D) Venules from BALB/c ESM-resistant mice. Linear regression lines are shown, with the inverse of the slope corresponding to convective flux. Data were analyzed using a general linear mixed model.

Finally, we sought to quantify changes in convective oxygen transport within the pial microcirculation post CM infection in both models. To test the hypothesis that convective transport was depressed in CM infected mice, we analyzed total O_2_ content as a function of convective flow, quantified using Poiseuille’s equation. As above, we compared groups using a general linear model, namely log(total O_2_ content) ~ *β*_0_ + *β*_1_ log(Q) + *β*_2_ [group] + *β*_3_ [log(Q):group], where Q is flow rate. These results are summarized in Fig. 3. In this analysis, we took 1/ *β*_1_ to represent convective or axial O_2_ flux, with *β*_2_ and *β*_3_ having the same interpretation as above. In the ECM-susceptible C57BL/6J strain, we found depressed convective O_2_ flux in arterioles in both CM groups compared with uninfected controls (p = 1.0 × 10^−15^), though no significant difference between early and late CM (interaction p = 0.0632). In arterioles from the ECM-resistant BALBc strain, we also found depressed convective oxygen flux (*β*_2_ p = 1.0 × 10^−15^, interaction p = 1.0 × 10^−15^). These analyses as well as those for venules are found in Fig. 3A, C and Fig. 3B, D, respectively.

## 4. Discussion

The principle finding of this study is that in both the ECM-susceptible (C57BL/6J) model and ECM-resistant (BALB/c) model of CM, convective and diffusive O_2_ flux along with pial hemodynamics are impaired. To our knowledge, this study is the first to quantify changes in diffusive (radial) and convective (axial) oxygen flux in models of CM using paired acquisition of perivascular and intravascular O_2_ tension and correlating these changes with global hemodynamic changes in Hb-O2 affinity. Furthermore, O_2_ transport appears to be more impaired in the ECM-resistant strain compared to the ECM-susceptible strain, as expected. It also appears that differences in oxygen transport and utilization were absent between early and late-stage CM C57BL/6J suggesting that alterations in pial microvascular transport either preclude worsening disease or are independent of symptoms. Our results further suggest that concomitant changes in p50, only 5 mmHg in the case of late-stage CM C57BL/6J mice, and oxygen diffusion result in insufficient O2 transport by the microcirculation and thus poor cerebral function. In adults with CM, intracerebral lactate production and cerebral spinal fluid lactate concentrations are increased6. These findings correlate with our results and can be explained by the impairment in cerebral microcirculation O2 transport, resulting from a combination of reduced perfusion, anemia, impaired RBC deformability and RBC sequestration. Lastly, the surprising reversibility of coma in experimental and clinical studies, which can last, in some cases, for several days, indicates that there is incomplete ischemia in CM.

Alterations in microvascular hemodynamics alone do not explain differences in the pathophysiological manifestation of CM, as supported by the few differences we observed in early vs. late-stage CM in the ECM-susceptible C57BL/6J model. Normally, the sigmoidal shape of the Hb-O2 promotes O2 delivery, provided the steep gradient for O2 delivery in the tissue from low tissue pO2. However, alterations in the Hb O2 affinity, in this case secondary to metabolic acidosis from systemic hypoxia and the Bohr effect, in conjunction with depressed radial O2 flux, as demonstrated in our study, impede tissue O2 delivery. In fact, based on our study, we posit that severe symptomatic CM requires both impaired microvascular O2 diffusion and impaired Hb-O2 kinetics. Although there is some evidence of autonomic nervous system dysfunction in malaria (Supanaranond et al., 1993), there is no evidence that neural control of vascular tone leads to impaired tissue perfusion. In CM, there seems to be a normal response of the cerebral resistance vessels (small arteries and arterioles) to changes in arterial O2 and CO2 (Warrell et al., 1988). Specifically, we observed that despite similar changes in microvascular diffusive and convective flux in early and late CM models, disease severity was different, likely due to the observed difference in p50.

Our results (Table 1) provide further insight the role of hypothermia in the pathogenesis of CM. Whether hypothermia induced by ECM is a protective mechanism or a strategy to preserve tissue function is still unknown. Hypothermia is known to decrease the metabolic rate of brain tissue, oxygen consumption9, and the growth rate of the malaria parasite (Rehman et al., 2016). Furthermore, our previous studies have demonstrated that hypoperfusion and subsequent ischemia is key to CM pathogenesis9. Although cerebral blood flow in CM is within the normal range, it is low in comparison with the arterial O2 content. Cerebral vascular resistance is increased, whereas cerebral O2 extraction is diminished. Yet, our results here suggest that concomitant microvascular hypoperfusion and decreased Hb-O2 affinity are required for severe disease. In this context, our results suggest that hypothermia is a compensatory mechanism to increase Hb-O2 affinity and is thus beneficial. Importantly, however, this analysis fails to account for the effect of the parasite on temperature regulatory centers in the brain. Thus, future studies are required for investigation of this hypothesis and specifically the role of hypothermia in CM outcomes.

Integral to this study is the use of robust statistical methods, namely simple linear model and ANCOVA, to both quantify and compare diffusive (radial) and convective (axial) oxygen flux and their relationship to Hb-O2 off-loading kinetics in the context of CM. Specifically, we introduced application of a general linear mixed model to log-log plots derived from experimental PQM data as a proxy to quantify flux, which more generally captures the non-linearities of oxygen diffusion in CM. In our calculation of convective flux, we assessed oxygen content as a function of microvascular flow, indicative of oxygen off-loading. Here, we demonstrated in both ESM-resistant and ESM-susceptible models impaired convective O2 post infection and similar degrees of O2 off-loading despite differing degrees of neurologic impairment. In fact, infected BALB/c died without neurologic symptoms of CM. Dynamic microvascular autoregulatory control of oxygen flow and diameter is necessary to maintain both the axial and radial flux of target tissues for sufficient oxygen perfusion. As alluded to above in the case of radial flux, we therefore hypothesize that neurologic sequalae from CM require both impaired microvascular oxygen transport and impaired Hb-O2 kinetics. In all cases, cerebral hypoxia ensues from the lack of a convective pO2 gradient across arterioles. However, so long as the sigmoidal Hb-O2 curve is maintained, then tissue hypoxia is at a minimum provided appropriate off-loading1,2,10. In the case when both the Hb-O2 is significantly right shifted and microvascular oxygen diffusion is impaired, then local regulatory mechanisms (i.e., modification of oxygen flow and arteriolar diameter) to maintain tissue pO2 fail to compensate. These results further suggest that intervening upon Hb-O2 kinetics by altering affinity may help preserve cerebral function despite impaired microvascular hemodynamics and oxygen transport. Future studies should therefore aim to investigate compounds with this effect, like 5-hydroxymethyl-2-furfural (5-MHF)26 and Voxelotor (GBT440) (Dufu et al., 2019), to test whether they have a protective effect in late-stage ESM despite impaired microvascular oxygen transport.

## 5. Conclusion

Impaired microcirculatory flow is essential in the pathophysiology of severe CM. Several factors combine to reduce microcirculatory flow-limiting convective transport of O2. Parasitized RBCs adhere to vascular endothelium, and the rigid adherent cells block the microvascular lumen, restricting the blood flow. In addition, the flowing infected RBCs are less deformable, increasing blood viscosity. This decreased number of functional capillaries during ECM is of great importance (Martini et al., 2007). There is a paradigm shift in understanding the pathogenesis of ECM mediated by microcirculatory dysfunction (decreased functional-capillary density and low O2 delivery), much earlier than macrocirculation dysfunction. Paradoxically, given that CM is believed to be a disease characterized by anemia, mitochondrial dysfunction, altered redox state, seizures, and impaired clearance by the liver, amongst other factors. Our experimental analysis suggests that CM can be attributed to microvascular dysfunction and decreased O2 delivery.

In conclusion, our study provides insight into the convective and diffusive fluxes in two different animal models of CM using statistical methods based linear mixed modeling. From our model applied to PQM data as a function of diameter and flow, we found that diffusive and convective fluxes were similarly impaired, yet those animals with more severe infection had significant shifts in Hb-O2 kinetics. We therefore hypothesize that concomitant impairment in microvascular O_2_ diffusion and Hb-O2 affinity is required for severe disease. Future studies should therefore aim to investigate whether altering Hb-O2 affinity pharmacologically has a protective effective despite impairment in microvascular oxygen diffusion.

## Figures and Tables

**Table 1:** Infected C57BL/6 (ECM-susceptible) and BALB/c (ECM-resistant) mice: Blood O_2_ characteristics, perivascular pH, and core temperature.

Group		Hematocrit, %	Hb, g/dL	Perivascular pH	P50^d^, mmHg	Parasitemia, %	Temperature, °C
**C57BL/6**	**Uninfected**	48 ± 3	15.6 ± 0.7	7.196 ± 0.008	41 ± 1	–	38.3 ± 0.8
	**Early ECM**	32 ± 3^a,b^	9.4 ± 0.6^a,b^	7.208 ± 0.012^b^	43 ± 1^b^	10.6 ± 3.3	35.3 ± 0.9^a,b^
	**Late ECM**	27 ± 2^a^	7.5 ± 0.7^a^	6.984 ± 0.009^a^	48 ± 2^a^	12.6 ± 3.4	33.1 ± 1.5^a^
**BALB/c**	**Uninfected**	51 ± 1	15.1 ± 0.5	7.120 ± 0.015	43 ± 1	–	37.1 ± 0.5
	**Infected**	32 ± 2^a,b^	9.3 ± 0.5^a,b^	7.153 ± 0.017^a,b^	44 ± 2^b^	10.5 ± 2.4	35.7 ± 0.8^a,b^

Values are means ± SD. Hb, hemoglobin concentration, P50, PO_2_ at which 50% of the Hb in the blood is saturated with O_2_.

a *P* < 0.05 compared with uninfected control;

b *P* < 0.05 compared with late ECM C57BL/6 (infected animals only);

c Measured at perivascular pH.

## Data Availability

Data will be made available on request.

## Availability of data and materials

The raw data and any materials supporting the conclusions of this article will be made available by the authors, without undue reservation, to any qualified researcher.
